# Effects of Accelerated Fermentation on the Chemical Composition and Quality of Beer

**DOI:** 10.3390/molecules31101695

**Published:** 2026-05-17

**Authors:** Marek Zdaniewicz, Szymon Lekowski, Aleksander Poreda, Robert Duliński

**Affiliations:** 1 Department of Fermentation Technology and Microbiology, Faculty of Food Technology, University of Agriculture in Krakow, Balicka Street 122, 30-149 Krakow, Polanda.poreda@urk.edu.pl (A.P.); 2 Centre for Innovation and Research on Prohealthy and Safe Food, University of Agriculture in Krakow, Balicka Street 104, 30-149 Krakow, Poland; 3 Department of Food Biotechnology, Faculty of Food Technology, University of Agriculture in Krakow, Balicka Street 122, 30-149 Krakow, Poland; robert.dulinski@urk.edu.pl

**Keywords:** beer fermentation, accelerating fermentation, chemical compounds, rotary jet head, mixing

## Abstract

The objective of this study was to examine the impact of using a rotary jet head (RJH) on the biosynthesis of byproducts of yeast metabolism and their role in shaping the flavor and aroma profile of bottom fermentation beer (lager style). The tests were conducted on an industrial scale, with fermentation in 3800 hL fermentation tanks. Experiments were conducted in a minimum of six replicates. The main quality indicators, including ethanol concentration and pH, were analyzed, along with key volatile compounds such as acetaldehyde, esters, higher alcohols, and DMS. Additionally, beer samples—both those fermented using forced mixing and those produced conventionally—were subjected to sensory evaluation. The study found that RJH did not cause changes in either the final ethyl alcohol concentration (6.74% in both samples) or the pH measurement results. The rotary jet head increased synthesis of certain volatile components, such as fusel alcohols by 5% and acetate esters by 14% for ethyl acetate and by almost 12% for isoamyl acetate. On the other hand, a more than threefold (8.23 to 2.54 mg/L) decrease in the undesirable acetaldehyde was observed in samples fermented with forced mixing. The resulting beers exhibited statistically significant differences in chemical composition; however, sensory analysis did not reveal these differences. This finding underscores the efficacy of the rotary jet head in expediting the beer production process without compromising its sensory quality.

## 1. Introduction

Beer is one of the most popular beverages consumed worldwide and the most frequently chosen alcoholic drink [1]. This is attributable to its organoleptic characteristics [2], which are predominantly influenced by the raw materials employed in the brewing process, namely water, malt, hops, and brewer’s yeast [3]. Yeast plays a particularly important role in shaping the aromas of beer [4,5], as it converts the sugars present in the wort into ethyl alcohol, carbon dioxide, and numerous volatile flavor and aroma compounds [6]. The finished beer’s bouquet may contain as many as 800 chemical compounds, but only a small fraction of them are directly responsible for the sensory experience during tasting [3]. The most significant components of yeast metabolism that influence the aroma of the finished beer include higher alcohols, aldehydes, esters, vicinal diketones, and sulfur compounds [7]. In addition, these compounds are predominantly regarded as undesirable at elevated concentrations, as they adversely impact the perception of the beer, thereby constituting a defect [6,8,9]. Consequently, the fermentation process and the judicious selection of its parameters are of paramount importance throughout the entire production process and pose a significant challenge for brewers [10,11].

The brewing industry is constantly driven by new technologies aimed not only at increasing competitiveness but also at optimizing the production process to reduce costs, energy consumption, and environmental impact [12]. In large-scale breweries, the use of HGB (high gravity brewing) technology is popular, as it allows for the production of a greater volume of finished beer in the same amount of time without the need to expand the brewery’s infrastructure [13]. However, fermentation is one of the most costly and time-consuming stages of beer production, mainly due to its impact on brewery production capacity and tank occupancy, which is why reducing its duration still yields significant savings [11]. The rate of fermentation is influenced by several parameters. As Kucharczyk et al. [14] assert, the temperature at which fermentation occurs is among the most significant factors. In addition to elevating the temperature, other factors that reduce fermentation time include increasing the initial yeast inoculum, enhancing oxygenation of the wort, and prolonging the filling time of the fermentation vessel. However, as the authors note, these processes have the potential to adversely affect yeast viability and, more significantly, modify the taste of the final product, which is the most critical factor considered by consumers [15].

Mixing the wort is imperative for effective fermentation, as it enhances yeast suspension and improves its interaction with wort components. However, in most cases, this is primarily caused by the agitation of the tank’s contents through the rising of carbon dioxide bubbles and temperature-related alterations [16]. There are methods for accelerating fermentation based on mechanical mixing [16] and for conducting continuous fermentation with yeast immobilized on a carrier [17,18], but these are not widely used in industry, and research is often conducted on a smaller scale.

When considering the aforementioned variables, it is imperative to achieve a balance between accelerating production (by reducing fermentation time) and preserving the desired quality of the final product [12]. In our previous study [19], we demonstrated the potential of mixing using a rotary jet head (RJH) to reduce fermentation and cooling times under industrial brewery conditions.

The objective of this study was to investigate, for the first time, how the intensification of the fermentation process using the RJH affects the concentrations of chemical compounds typical of lager beer. Concurrently with the monitoring of volatile compounds, the pH value was measured on subsequent days of the process, and the sensory quality of the finished beer was assessed relative to a control sample. The study, conducted on an industrial scale, sought to address the question of whether beer produced using an innovative tank-mixing system (RJH) differs in chemical composition and sensory characteristics from beer produced without the introduced process modification.

## 2. Results and Discussion

### 2.1. Ethyl Alcohol

Ethanol is the second most important metabolite, after carbon dioxide, produced by yeast during fermentation [1]. It is formed by the conversion of glucose to pyruvic acid, which is then converted to acetaldehyde by pyruvate decarboxylase. Subsequently, acetaldehyde is reduced to become ethanol [20]. The concentration of ethyl alcohol is directly related to the original wort extract, but may vary depending on factors such as the raw materials used or mashing parameters [21]. The declared ethanol content in beer should be consistent with the style in which it was produced. Beers with an alcohol content between 4.5 and 6% *v*/*v* are most common [22,23], but there are also beer styles that may have a higher level of this component [24].

**Figure 1:**
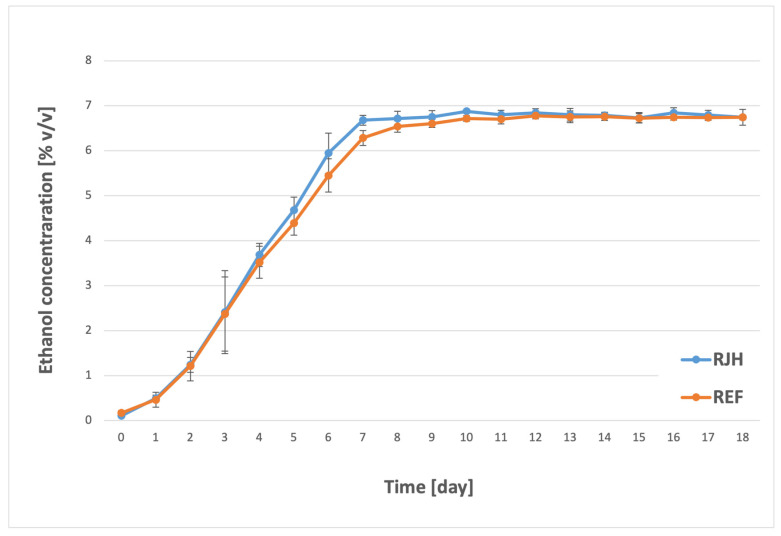
Ethyl alcohol concentration over the course of the fermentation and maturation process with (RJH) or without (REF) mixing (*n* ≥ 6; mean ± standard deviation, *p* < 0.05).

In the case of the forced-mixing fermentation under study (Figure 1), it was observed that, in all samples, the yeast synthesized ethyl alcohol at a similar level through the fourth day of the process. The initial substantial discrepancies were discovered on the fifth day of fermentation, when the alcohol concentration in the forced-mixing tank (RJH) reached 4.7% *v*/*v*, in contrast to 4.4% *v*/*v* for the control sample, which was devoid of mixing (REF). The observed differences can be explained by the premature sedimentation of yeast cells in the tank without mixing, as previously observed by Boulton [25]. From that day onward, increased ethanol synthesis was observed in the tank equipped with a rotary jet head. On the seventh day of the process, an average result of 6.7% *v*/*v* ethanol was obtained for the agitated samples and 6.3% *v*/*v* for the control samples. A comparison of the results from the seventh day for RJH and the results from the eighth day for REF reveals that they did not differ significantly. The results for the subsequent days and the final alcohol concentration in both samples demonstrate a similar pattern. This finding suggests that the process progressed more rapidly in the tank with forced mixing (RJH), yet the final concentration of the examined component remained constant for both samples. The absence of statistically significant differences suggests that the yeast utilized all the available extract, though this process occurred more rapidly in the tank equipped with a rotary jet head. Consequently, RJH does not affect the final ethanol concentration; rather, it reduces the time required to reach it by approximately 24 h. The findings of the present study are consistent with those of other researchers investigating the effect of forced mixing (using methods other than RJH) during the fermentation process. Nienow et al. [16] investigated the effect of mechanical mixing on the efficiency of the brewing process, including that of Grolsch Lager beer. They demonstrated that such mixing accelerates the fermentation process by approximately one day, but without a significant impact on final fermentation, and consequently on the final alcohol concentration. Similar conclusions had previously been reached by Boswell et al. [26], who also found no effect of the applied mixing on the final ethanol content in beer. This phenomenon was attributed to the nutrients available to the yeast, rather than to the intensity of the process itself. The accelerated attainment of the final ethyl alcohol level in a fermentation tank equipped with a rotary jet head may be related to the facilitated contact between the yeast and the wort components. During standard fermentation, without the implementation of a mixing process, the yeast settles to the bottom of the tank [27]. This facilitates the subsequent removal and reuse of the yeast. However, it also hinders the yeast’s access to the fermentable sugars present in the wort. In the case of RJH, this problem is minimized, as continuous mixing maintains a homogeneous distribution of the tank contents throughout the process. This does not affect the final degree of fermentation, as the amount of nutrients available to the yeast remains unchanged; rather, it facilitates access to these nutrients. The results obtained are of particular importance in the context of large breweries, which are often part of international brewing groups [28], for which quality, and in particular its maintenance, is paramount.

### 2.2. pH

**Figure 2:**
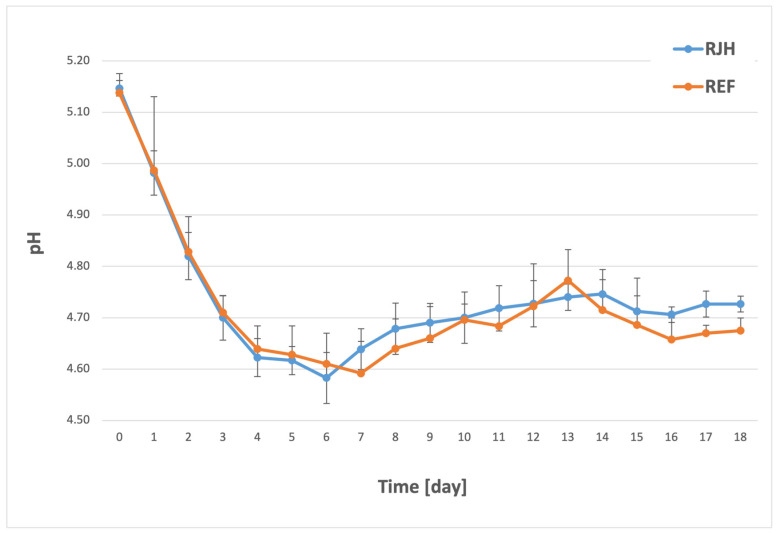
Changes in pH over the course of the fermentation and maturation process with (RJH) or without (REF) mixing (*n* ≥ 6; mean ± standard deviation, *p* < 0.05).

The pH is a pivotal factor in determining the stability and quality of beer during storage, along with ethanol, carbon dioxide, and low residual sugar levels, among other elements. A low pH has been shown to inhibit microorganisms by affecting enzymatic activity and enhancing the antiseptic properties of hops [29]. The pH value may exhibit variation within a given style, but it generally ranges from 3.4 to 4.7 [30]. Figure 2 shows pH changes for a tank equipped with a rotary jet head and for a control tank without forced mixing.

A significant decrease in pH was observed in both samples starting from the first day of fermentation. The lowest recorded pH level, slightly below 4.6, was observed on the sixth day for RJH and on the seventh day for REF. In the ensuing days, fluctuations in pH ranging from +/−0.1 were observed, though these variations were not deemed to be statistically significant. Consequently, the pH level was reduced to the optimal range (maximum of 4.8) for both the forced-mixing sample and the control sample, as desired by the plant. The use of a rotary jet head did not exert a substantial influence on the pH values attained during the fermentation process. Boswell et al. arrived at analogous conclusions [26]. Using an alternative mixing technique, it was determined that the effect on pH formation was negligible. The results obtained are of significant importance in the context of maintaining the high quality and reproducibility of the beer produced, in which pH not only influences better antimicrobial properties but may also affect foam stability [31] or yeast flocculation [32]. This latter characteristic may be of particular importance in the context of RJH use, as rapid sedimentation is crucial after mixing is complete in order to collect the yeast sediment and begin cooling.

### 2.3. Acetaldehyde

Acetaldehyde is the most important carbonyl compound produced during alcoholic fermentation. Yeast synthesizes it from pyruvate, and it serves as an intermediate in ethanol production. This process is catalyzed by the enzyme pyruvate decarboxylase [33]. The concentration of acetaldehyde in beer can range from 1 to 20 mg/L [8], although it is typically found at relatively low levels due to the efficient reduction of the substance by yeast during fermentation [34]. The detection threshold for this compound is approximately 10–20 mg/L [3]. At lower concentrations, it can impart pleasant green apple aromas, but at higher levels, it is associated with notes reminiscent of emulsion paint [34]. It is cited as one of the most significant factors influencing the quality of the finished beer, not only due to its characteristic aromas but also its impact on the product’s shelf life during storage [35]. Consequently, it is imperative to meticulously monitor and maintain its concentration in beer, ensuring it remains at a relatively low level.

**Figure 3:**
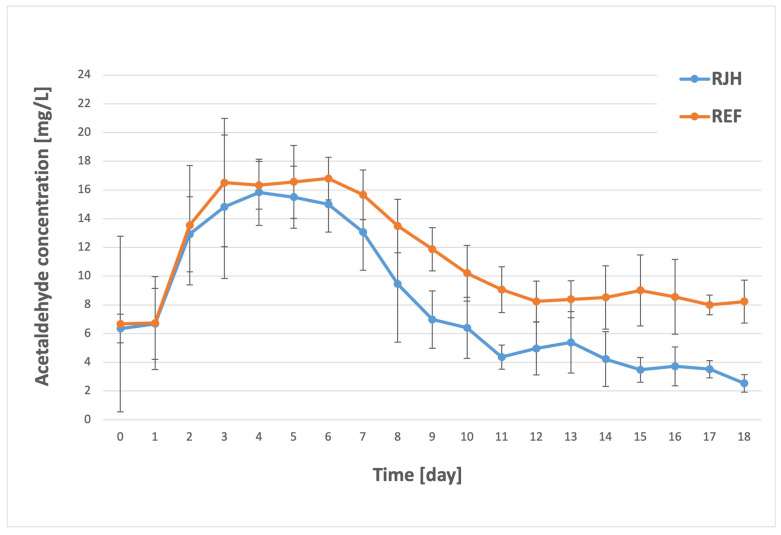
Acetaldehyde concentration over the course of the fermentation and maturation process with (RJH) or without (REF) mixing (*n* ≥ 6; mean ± standard deviation, *p* < 0.05).

An analysis of the results indicates that the synthesis of acetaldehyde commenced after a single day of fermentation. The highest observed concentration occurred during days 3–6 of the process, ranging from 14.83 to 15.84 mg/L for the tank with forced mixing and from 15.67 to 16.79 mg/L for the tank without mixing (Figure 3). The process of aldehyde synthesis exhibited a modest increase in efficiency when employing a rotary jet head. The highest recorded concentration (15.84 mg/L) was observed on the fourth day of the process (RJH), whereas in the control sample (REF), it did not occur until the sixth day and exhibited a slightly higher value (16.79 mg/L). While the observed differences were not statistically significant, a significantly faster reduction in acetaldehyde occurred in the tank with mixing starting from the sixth day. On the eleventh day, the concentration of the tested component in the sample with the rotary jet head was 4.37 mg/L, and this concentration remained at a similar level until the cooling stage. Conversely, for the non-mixed tank, the concentration measured on the twelfth day was 8.25 mg/L. The differences in the final acetaldehyde content in beer produced with and without the RJH were statistically significant. It is noteworthy that the studied component fell within the brewery’s prescribed specification range (0–7 mg/L) for the tank equipped with a rotary jet head. In contrast, for the tank devoid of a rotary jet head, the component’s concentration fell within the permissible tolerance range (7–9 mg/L). The observed onset of aldehyde synthesis as early as the initial days of fermentation is consistent with information reported in the literature [36,37], according to which yeast produces the most aldehyde during the growth phase. In their study, Pires et al. [17] conducted research on the effect of continuous fermentation on the quality of the beer produced. For one variant of Pilsner beer, they demonstrated that aldehyde production was half as high in beer produced via continuous fermentation as in beer produced using traditional methods. This corresponds with the results we obtained—for RJH, the final concentration of the tested compound is nearly half that of standard fermentation. In addition, Kucharczyk and Tuszyński [38] investigated the effect of fermentation temperature on the production of acetaldehyde by lager yeast. According to their study, increasing the temperature from 10 °C to 11.5 °C resulted in a decrease in the final concentration of acetaldehyde in the beer, without affecting the sensory perception of the product due to other yeast metabolites.

### 2.4. Higher Alcohols

**Figure 4:**
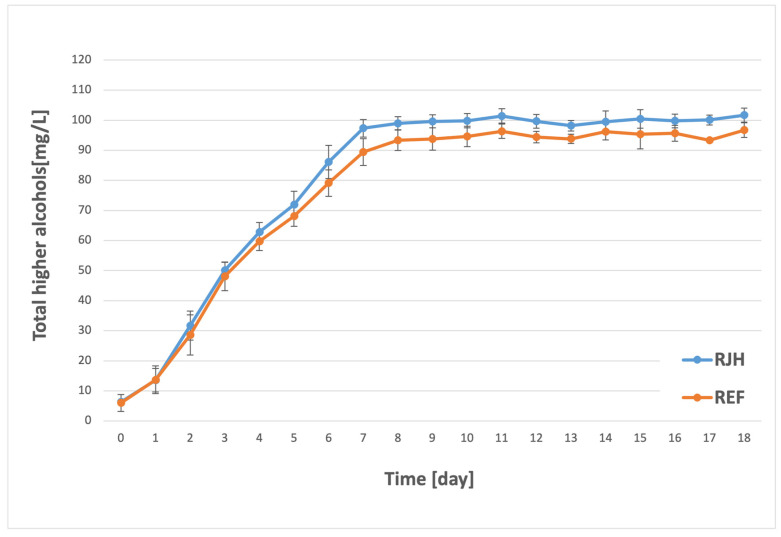
Fusel alcohol concentration (sum of propanol, isobutanol, and amyl alcohol concentrations) over the course of the fermentation and maturation process with (RJH) or without (REF) mixing (*n* ≥ 6; mean ± standard deviation, *p* < 0.05).

The group of higher alcohols, often referred to as fusel alcohols, consists of compounds that are important for shaping the aroma of beer [39]. Their contributions can be both positive and negative. At higher concentrations (above 300 mg/L), they produce sharp and unpleasant odors and flavors [34]. Moreover, their significance extends to their role in the formation of other chemical compounds, specifically esters [40]. The production of fusel alcohols is closely linked to the metabolism of amino acids, which serve as a nitrogen source for yeast. The biosynthesis of higher alcohols is initiated by the decarboxylation of α-keto acids to aldehydes, which are subsequently reduced to the corresponding alcohols. It is noteworthy that α-keto acids can be formed via two distinct pathways: the catabolic Ehrlich pathway and the anabolic pathway [3]. The most significant compounds in this category include propanol, isobutanol, amyl alcohol, and phenylethanol [1,41]. In the present study, the concentrations of all the fusel alcohols listed above were summed and presented in a single graph (Figure 4).

For both tanks, the synthesis of higher alcohols began at an early stage of fermentation. A substantial increase in concentration was observed until approximately the seventh or eighth day of the process. The maximum fusel alcohol concentration for both samples was nearly equivalent to the standard of the plant, which ranges from 0 to 100 mg/L. Marginally higher levels were documented in the context of fermentation with forced mixing. Consequently, the implementation of the rotary jet head resulted in an augmentation in the production of higher alcohols, yet their concentrations remained within the brewery’s acceptable parameters. Preliminary research has indicated that forced mixing may result in elevated levels of fusel alcohol production. However, these studies were conducted on a significantly smaller scale, more akin to laboratory experiments. Boswell et al. [26] conducted a study that revealed an increase in the concentration of higher alcohols in fermentations that were conducted using a mixing process. Concurrent findings were posited by Nienow et al. [16], whose study endorsed the implementation of mixing on a broader scale. However, their tests were conducted on a macro scale. Furthermore, higher levels of this component were detected in samples of one of the Pilsner variants that underwent continuous fermentation in a reactor by Pires et al. [17]. However, the results of our study, conducted on the scale of a large industrial brewery, suggest that the use of a rotary jet head during the mixing process has no significant effect on the final concentration of higher alcohols in beer. While a slight increase was observed, it was not statistically significant compared to the control samples.

### 2.5. Esters

**Figure 5:**
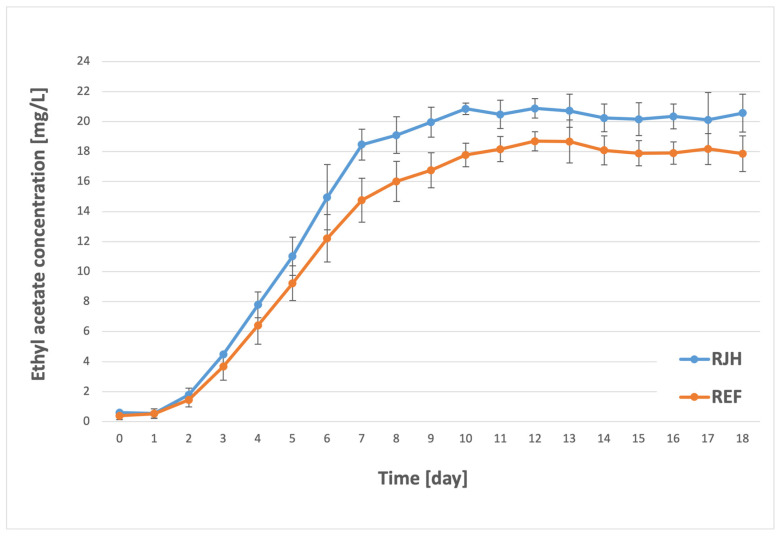
Ethyl acetate synthesis over the course of the fermentation and maturation process with (RJH) or without (REF) mixing (*n* ≥ 6; mean ± standard deviation, *p* < 0.05).

**Figure 6:**
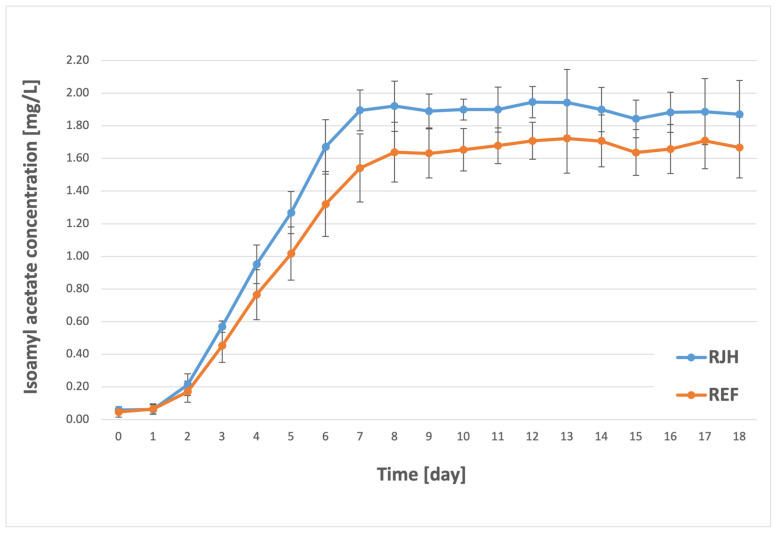
Isoamyl acetate synthesis over the course of the fermentation and maturation process with (RJH) or without (REF) mixing (*n* ≥ 6; mean ± standard deviation, *p* < 0.05).

Esters are formed during an enzyme-catalyzed condensation reaction between alcohols and acids [6,42]. These compounds are among the most volatile components in beer. At lower concentrations, they can impart pleasant floral and fruity aromas and shape the beer’s profile; however, at higher concentrations, they impart unpleasant, dominant solvent notes [34,43]. Among the most significant esters are two esters of acetic acid: ethyl acetate, which contributes fruity and solvent-like aromas, and isoamyl acetate, which imparts banana–pear notes [44]. The concentrations of these two compounds in beer vary significantly. The detection threshold for ethyl acetate is reported at 30 mg/L, whereas for isoamyl acetate, it is only 2 mg/L [3]. However, these compounds typically occur at lower levels and do not significantly contribute to the deterioration of the finished beer’s aroma [45]. In the present study, the two esters previously mentioned were the focal point, and their concentrations are presented below (Figure 5 and Figure 6).

Analysis of the results obtained for ethyl acetate revealed that a significant increase in its synthesis began on the second day of the process. A notable effect of the rotary jet head on ester production was observed starting on the fifth day. For the RJH tank, the concentration was 11 mg/L, while for the REF tank it was 9.2 mg/L. The maximum ethyl acetate content in the beer was measured on the ninth day of fermentation for the sample with forced mixing (20.9 mg/L) and only after three additional days for the sample without mixing (18.7 mg/L). In both cases, the concentration remained relatively constant until the conclusion of the process. It is noteworthy that the final concentration of ethyl acetate in both the RJH and REF tanks fell within the plant’s specified range of 15–25 mg/L. In the case of isoamyl acetate, a gradual increase in concentration was also observed from the initiation of fermentation until the seventh/eighth day of the process (after which it stabilized at a similar level). During the initial three-day period, the content of isoamyl acetate exhibited no substantial variation among the samples. A statistically significant difference was not observed until the fourth day, with a 0.2 mg/L higher concentration for RJH. In the subsequent days, the synthesis of isoamyl acetate was expedited in the tank with forced mixing, reaching a maximum concentration of 1.9 mg/L. For the control sample, the highest recorded concentration was approximately 10% lower, amounting to 1.7 mg/L. It should be noted that the content and rate of formation of the studied component were significantly dependent on the use of the rotary jet head. However, in both cases, the final concentration fell within the brewery’s standards, which were set at a maximum of 2.5 mg/L. The results obtained in this study are not consistent with those reported by other researchers concerning the application of mixing during the fermentation process. It must be acknowledged that the aforementioned studies were conducted on a significantly smaller scale and employed a distinct mixing method. However, it is noteworthy that both Boswell et al. [26] and Nienow et al. [16] observed a decrease in ester production in their analyses of samples that underwent mixing during the fermentation process. Conversely, Pires et al. [17], while researching continuous fermentation, measured a decrease in the final concentration of the two esters: ethyl acetate and isoamyl acetate. A notable finding among the authors mentioned above is the observation of an increase in the concentration of higher alcohols in the test samples. However, it should be noted that ester production in beer is also influenced by other factors, including wort composition, fermentation temperature, and the yeast strain used [7,38].

### 2.6. DMS

**Figure 7:**
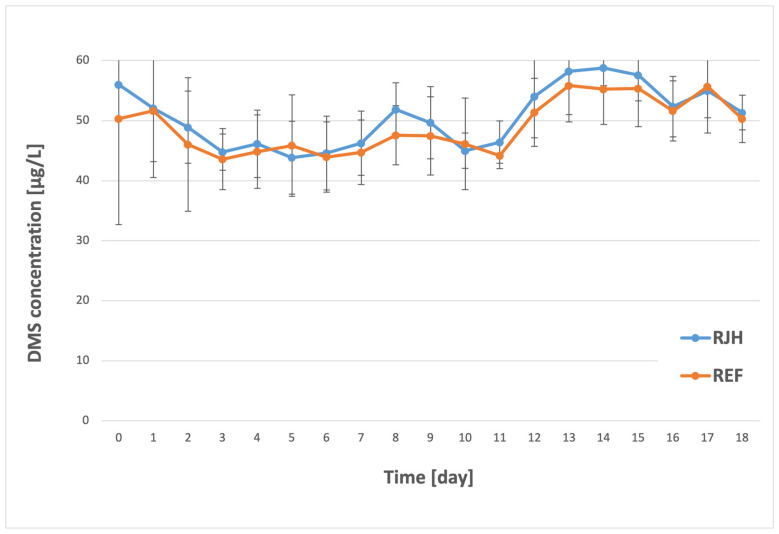
DMS concentration over the course of the fermentation and maturation process with (RJH) or without (REF) mixing (*n* ≥ 6; mean ± standard deviation, *p* < 0.05).

Dimethyl sulfide is the best-known sulfur compound responsible for the aroma of beer [46]. At lower concentrations, it contributes to the aroma of bottom-fermented beers [47]. However, its detection threshold is quite low (30–100 µg/L). Exceeding this threshold can result in aromas reminiscent of corn, cooked vegetables, or tomato sauce [34,46]. This compound can be formed in beer via two main pathways. First, its precursor, S-methylmethionine, is a non-protein, thermolabile amino acid that is synthesized during grain malting. DMS may also be formed during fermentation from dimethyl sulfoxide (DMSO) as a yeast metabolite [8,34]. Changes in dimethyl sulfide concentration for rotary jet head fermentation and the control sample are illustrated in Figure 7 below.

A review of the results obtained reveals that, during the initial phase of the process (the first three days of fermentation), the concentration of the tested compound decreased from 56.0 to 44.8 µg/L (RJH) and from 51.6 to 43.60 µg/L (REF). It is noteworthy that the obtained concentrations do not differ significantly from one another; however, due to the higher initial DMS content in the sample with forced mixing compared to the control sample, the greater absolute reduction in dimethyl sulfide observed in the RJH sample was associated with its higher initial concentration. In the subsequent days, there was no statistically significant change in the DMS content. An increase in the tested component was observed on the thirteenth day, reaching 58.3 µg/L for the mixed tank and 55.9 µg/L for the control sample. The measured values did not differ significantly and fluctuated to a greater or lesser extent in the following days. It should be highlighted that, consistent with the findings for the other chemical compounds under analysis, DMS also remained well below the plant’s specified limit of 70 µg/L. Given the above, it can be concluded that the use of a rotary jet head does not significantly affect the excessive production of dimethyl sulfide. An initial decrease in the concentration of this organoleptically important compound was observed for both samples. This can be attributed to vigorous fermentation, during which carbon dioxide is released, leading to the formation of bubbles that partially remove this highly volatile component [46]. The subsequent increase in DMS content is most likely due to the transformation of DMSO, which is reduced during fermentation [48]. Unfortunately, the available literature on forced mixing does not address the issue of dimethyl sulfide. The previously cited studies also focused solely on other compounds that have already been analyzed. Therefore, this study offers novel insights into the understanding of accelerated fermentation and its impact on specific organic compounds present in beer.

### 2.7. Sensory Analysis

**Figure 8:**
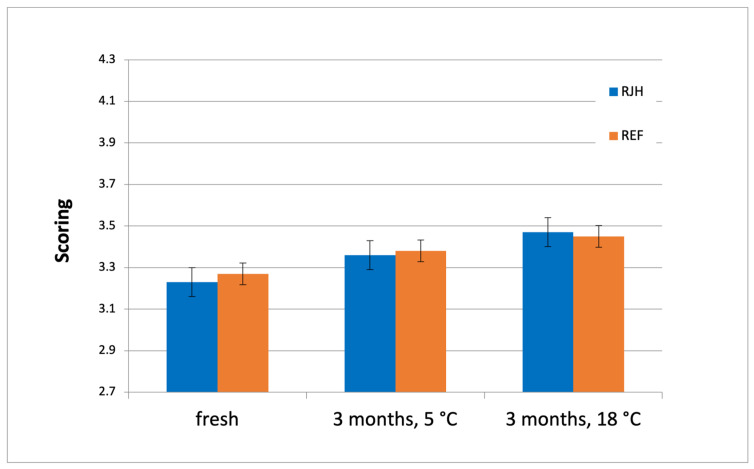
Results of the sensory analysis of beers depending on the storage conditions of the finished product (*n* ≥ 6; mean ± standard deviation, *p* < 0.05).

**Figure 9:**
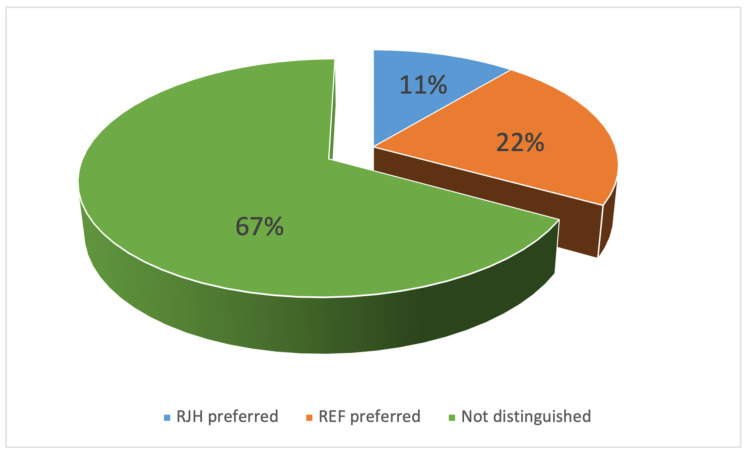
Results of the beer recognition test (triangle test) (*n* ≥ 6; *p* < 0.05).

Since time immemorial, the human senses have been the primary means of assessing the quality and safety of food. Product characteristics such as taste, smell, and appearance made it easy to determine whether food or beverages were fit for consumption [49]. Today, sensory analysis plays a key role in the food industry and is used for quality control, among other things [50]. In some cases, it may be deemed more crucial than chemical and physicochemical analyses, as it emphasizes consumers’ perceptions and their assessment of product quality. The perception of a similar concentration of the same compound can vary significantly between different beers. Consequently, the use of tasting panels of trained specialists is imperative to discern subtle variations among samples [51]. As part of the study, a sensory evaluation was conducted on beer that underwent fermentation using a rotary jet head (RJH) and on a control beer (REF). Samples of fresh beer and beer stored under various conditions for a period of three months were analyzed. The figures below (Figure 8 and Figure 9) illustrate the results.

Sensory scoring

Triangle test

There are some compounds that could have a negative influence on the beer aroma and quality, including during storage. These compounds include aldehydes, such as acetaldehyde [38]. Fermentation using RJH showed a greater reduction in acetaldehyde during the process (Figure 3). Acetate esters are one of the most important flavor and aroma compounds [40]. Concentrations of these compounds varied depending on the fermentation method (Figure 5 and Figure 6). However, the evaluation results for both fresh beer and beer stored for three months at both temperatures did not differ significantly. This indicates that both beers—those produced using a rotary jet head as well as the control sample—received equivalent ratings during the tasting panel. The use of RJH did not show a significant impact on the deterioration of the quality of the beer under analysis. Moreover, both tasting samples received high scores (the range of 3.0–3.3 indicates good quality beer). It should be highlighted that, according to the plant’s internal specifications, an ideal beer is one that receives a score close to 2.7. The worst possible score is 4.3. In the triangle test, the tasting panel consisted of nine professional tasters tasked with determining whether beers produced using a rotary jet head differed sensorily from the control samples. Only three people (33% of the tasters) were able to distinguish the RJH beer from the REF beer. Of this group, one person preferred the RJH sample, while the other two preferred the control sample. The remaining six people were unable to identify which sample was the control and which was the beer produced using forced mixing. These findings may also be influenced by the beer matrix, as it is known that matrix composition can modulate flavor perception and sensory thresholds. Ali et al. [52] conducted a sensory analysis of date vinegar-based beverages using a panel of experts and consumers, as well as an e-tongue. During the analysis, several key quality attributes were evaluated, similarly to this study. They emphasized that, despite differences in the concentrations of individual aroma compounds, sensory evaluation is crucial, as it allows for the determination of a beverage’s acceptance by consumers. Kucharczyk et al. [53] researched the effect of wort aeration on fermentation and beer quality. In line with this publication, they demonstrated that, despite significant differences in the content of volatile compounds in the beers studied, these variations did not significantly affect the sensory quality of the final product. It is noteworthy that beers aged for three months (under both refrigerated and near-room temperature conditions) received lower ratings than fresh beers. This phenomenon is attributable to the occurrence of processes in the finished product related to, among other things, oxidation, degradation of bitter compounds [54], and exposure to UV radiation [55].

## 3. Materials and Methods

### 3.1. Materials

#### 3.1.1. Wort

All-malt lager wort was produced in 1000 hL batches at the brewery where the experiments were conducted. It was hopped with Magnum extract for bitterness at the beginning of boiling and with Marynka for aroma at the end of the boiling stage. Each fermenter—the RJH tank and the control tank (REF)—was filled with three batches. The wort was made from 100% barley malt using HGB technology (adjustable extract: 15° Plato). It contained approximately 260 mg/L of free amino nitrogen (FAN) and had a dissolved oxygen level of 10 mg/L prior to fermentation.

#### 3.1.2. Yeast

Fermentation was carried out using third-generation, bottom-fermenting yeast, *Saccharomyces pastorianus* W 34/70. The pitching rate was 7 × 10^6^ cells/mL.

### 3.2. Fermentation

Fermentation was conducted in two identical stainless steel tanks, each equipped with three temperature sensors at different heights and a small pump for sampling. The entire process lasted approximately 18 days and consisted of fermentation (10 °C), maturation (14.5 °C), and lagering (−0.7 °C). The main fermentation process continued until the extract reached 7.8°Plato. Then, the temperature was raised to 14.5 °C, and the beer was matured until it had the appropriate level of vicinal diketones. After that, it was cooled to −0.7 °C to achieve entry clarity. The temperature was controlled by a tank cooling system using glycol and monitored by three temperature sensors (Pt100), with which each tank was equipped.

### 3.3. Experimental Procedure

The research was conducted under industrial conditions, in accordance with Zdaniewicz et al. [19]. A rotary jet head (Alfa Laval, Ishøj, Denmark) was installed in a cylindrical–conical tank with a total capacity of 3800 hL (working capacity 3200 hL). The pipeline entered the tank through the conical section, as recommended by the manufacturer. The rotary jet head was positioned approximately 3.5 m above the base. An 11 kW pump operated at 6.4 bar and 40 Hz throughout the process, resulting in a flow rate of 290 hL/h.

### 3.4. Analytical Methods

#### 3.4.1. Sampling Procedure

Samples (approx. 500 mL) were taken daily using small pumps installed in the tanks. Each sample was then mixed with 4 g of diatomaceous earth and filtered through a filter paper until approx. 300 mL of filtrate was obtained. The filtrate was then heated to 20 °C and degassed for 20 min on a universal shaker (150 rpm).

#### 3.4.2. Ethyl Alcohol and pH

The measurements were performed using the Alcolyzer wort and beer analyzer (Beer Analyzer DMA 4500+ Anton Paar, Warsaw, Poland). The degassed sample was filtered a second time, then poured into two 50 cm^3^ cups that had been placed in the Alcolyzer. The concentration of ethyl alcohol was determined by measuring the test liquid’s near-infrared (NIR) spectrum. Concurrently with the alcohol measurement, the device performed a pH measurement.

#### 3.4.3. Volatile Compounds

The concentrations of selected volatile components were measured using a GC 8000 gas chromatograph (Fisons Instruments, Ipswich, UK) equipped with a flame ionization detector (FID) integrated with an HS 800 autosampler. The injection of the sample was facilitated by means of an autosampler, with the sample preheating temperature set at 40 °C for a duration of 40 min. The injection volume was designated as 0.75 mL in the splitless mode, and the autosampler syringe temperature was maintained at 60 °C. The instrument was operated under the following conditions: the program temperature was set at 45 °C for 10 min, followed by a rise at a rate of 5 °C per minute to 120 °C. This temperature was maintained for 8 min and then decreased to 45 °C at a rate of 15 °C per minute. The injection zone temperature (injector) was set to 140 °C, the carrier gas was helium at a pressure of 65 kPa, and the flow rate was set to 4–6 mL per minute. The quantitative concentrations were calculated using a computer program based on the calculated peak areas.

A 2.5 mL sample of centrifugally filtered beer was placed into a vial, after which an internal standard (a mixture of 3-pentanol and n-butanol) was added. Following equilibration of the solution (composition equalization), vapors were extracted from the closed space above the sample using a syringe and introduced into the injection chamber of the gas chromatograph. The separation was carried out using a DB–WAX capillary column (l = 60 m; d = 0.53 mm; f = 1 µm) packed with a highly polar stationary phase (cross-linked polyethylene glycol).

### 3.5. Sensory Analysis

#### 3.5.1. Sensory Scoring

Sensory analysis of the beer was conducted following the brewery’s standard procedures in the form of an anonymous questionnaire containing the evaluated attributes, as part of a project investigating the impact of a rotary jet head on beer quality. The evaluation involved comparing each sample with a control sample using a profile test. The test assessed 12 beer characteristics, including ester and hop aroma, bitterness, sulfur compounds, sweetness, sourness, fullness, balance, and aftertaste. The analysis was performed by a team of nine specially trained tasters, all employees of the brewery. The final result for each beer was calculated as the arithmetic mean of the scores obtained during tasting. According to the brewery’s internal criteria, lower scores indicate better quality, with 2.7 being the best possible score and 4.3 the worst. The scale of scoring was as follows: very good (2.7–3.0), good (3.0–3.3), neither good nor poor (3.3–3.7), poor (3.7–4.0) and very poor (4.0–4.3).

#### 3.5.2. Triangle Test

To assess subtle or no differences between the beer produced using the rotary jet head and the control sample, a triangle test was conducted according to the EBC method: 13.7 Sensory Analysis—Triangle Test. Each member of the sensory panel received three coded samples (two identical and one different). In addition, each assessor was asked to indicate their preferred beer.

### 3.6. Statistics

The results are presented as the mean of six or more independent experiments, with bars representing the standard deviation. The data were analyzed using one-way analysis of variance (ANOVA). The significance of the difference for each parameter was analyzed separately using Duncan’s range test, *p* < 0.05 (Statistica v. 10, StatSoft Inc., Krakow, Poland).

## 4. Conclusions

The use of a rotary jet head during fermentation affects the synthesis of certain volatile compounds. The study revealed a decrease in acetaldehyde content and increased production of esters, i.e., ethyl acetate and isoamyl acetate, as well as a slight increase in the content of higher alcohols. Despite the observed differences in the final concentrations of these compounds, no effect of forced mixing on the sensory characteristics of the beer was demonstrated. The use of the RJH also did not alter the final ethyl alcohol content and had no effect on the pH achieved during fermentation. Based on the present study, as well as our previous research, it can be concluded that the use of a rotary jet head in the beer production process is justified not only by time and energy savings (shortening fermentation and cooling), but also by the absence of significant changes in the final product.

## Data Availability

The data are contained within the article.
